# Tanycytes from a bird’s eye view: gene expression profiling of the tanycytic region under different seasonal states in the Svalbard ptarmigan

**DOI:** 10.1007/s00359-024-01716-3

**Published:** 2024-09-20

**Authors:** Daniel Appenroth, Alexander C. West, Shona H. Wood, David G. Hazlerigg

**Affiliations:** Arctic Seasonal Timekeeping Initiative (ASTI), Arctic Chronobiology & Physiology, Arctic & Marine Biology, BFE, UiT - Arctic University of Norway, Tromsø, Norway

**Keywords:** Tanycytes, Seasonal, Photoperiod, Metabolism, Birds

## Abstract

In mammals and birds, tanycytes are known to regulate thyroid hormone conversion, and this process is central to the control of seasonal reproduction. In mammals, this cell type is also implicated in retinoic acid signalling, neurogenesis, and nutritional gatekeeping, all of which have been linked to hypothalamic regulation of energy metabolism. Less is known about these potential wider roles of tanycytes in birds. To address this gap, we combined LASER capture microdissection and transcriptomics to profile the tanycytic region in male Svalbard ptarmigan, a High Arctic species with photoperiod-dependent seasonal rhythms in reproductive activation and body mass. Short photoperiod (SP) adapted birds were transferred to constant light (LL) to trigger breeding and body mass loss. After five months under LL, the development of photorefractoriness led to spontaneous re-emergence of the winter phenotype, marked by the termination of breeding and gain in body mass. The transfer from SP to LL initiated gene expression changes in both thyroid hormone and retinoic acid pathways, as described in seasonal mammals. Furthermore, transcriptomic signatures of cell differentiation and migration were observed. Comparison to data from Siberian hamsters demonstrated that a photoperiod-dependent re-organisation of the hypothalamic tanycytic region is likely a conserved feature. Conversely, the spontaneous development of photorefractoriness showed a surprisingly small number of genes that reverted in expression level, despite reversal of the reproductive and metabolic phenotype. Our data suggest general conservation of tanycyte biology between photoperiodic birds and mammals and raise questions about the mechanistic origins of the photorefractory state.

**Supplementary Information:**

The online version contains supplementary material available at 10.1007/s00359-024-01716-3.

## Introduction

Temperate and polar latitudes are characterised by yearly cycles in daylength, temperature and nutrient availability, known as the seasons. Animals inhabiting those seasonal habitats synchronise their behaviour, physiology and metabolism to the annual cycle. For example, birds and mammals breed seasonally to ensure that the raising of young coincides with the favourable nutritional conditions of spring and summer, allowing the energy demands of the juvenile period to be met. The change in photoperiod (daily duration of the light phase) is the primary environmental signal by which seasonal animals anticipate upcoming seasons and synchronise their life cycles accordingly. To do so, the environmental photoperiod must be perceived, integrated, and then ultimately trigger transitions between seasonal phenotypes. The photoreceptive pathways involved in photoperiodic synchronisation differ between birds and mammals, with the former employing deep encephalic photoreception (Halford et al. 2009; Nakane et al. 2010; Pérez et al. 2019)⁠ while the latter employs a retina-suprachiasmatic nucleus-pineal melatonin relay pathway (Dardente et al. 2010; Masumoto et al. 2010; Wood et al. 2020)⁠. For comparative review, see (Hazlerigg and Loudon 2008; West and Wood 2018; Nakane and Yoshimura 2019)⁠⁠.

Despite this, there is remarkable conservation in the downstream pathways of photoperiodic integration (Yoshimura et al. 2003; Hanon et al. 2008; Hazlerigg and Loudon 2008; Nakane et al. 2013)⁠⁠⁠. Transitions to long photoperiod (LP) trigger the expression of thyroid-stimulating hormone subunit beta (*Tshb*) and its release from the *pars tuberalis* (PT) of the anterior pituitary gland (Hanon et al. 2008; Nakao et al. 2008a)⁠⁠. PT-derived TSH binds to its receptor expressed in specialised cells lining the third ventricle, known as tanycytes (Horstmann 1954)⁠, triggering a cAMP-dependent pathway ultimately leading to increased expression of type II iodothyronine deiodinase (*Dio2*) (Hanon et al. 2008; Nakao et al. 2008a; Ono et al. 2008)⁠⁠. DIO2 locally converts thyroxine (T4) into triiodothyronine (T3) by outer ring deiodination, leading to increased hypothalamic concentration of bioactive thyroid hormone under LP. Conversely, short photoperiod (SP) coincides with increased expression of type III iodothyronine deiodinase (*Dio3*) within the tanycytes causing thyroid hormone deactivation (Yasuo et al. 2005; Nakao et al. 2008b; Sáenz de Miera et al. 2013; Milesi et al. 2017)⁠⁠.

In birds and long-day breeding mammals, increased T3 levels in the hypothalamus lead to increased release of gonadotropin hormone-releasing hormone (GnRH), ultimately causing increased release of gonadotropins from the anterior pituitary and gonadal activation under LP (Yoshimura et al. 2003; Yamamura et al. 2004, 2006; Henson et al. 2013; Klosen et al. 2013; Dardente et al. 2014; Quignon et al. 2020)⁠⁠. Contrarily, in short day-breeding mammals (e.g. sheep) increased T3 availability under long days leads to decreased GnRH release and gonadal quiescence (Hanon et al. 2008; Sáenz de Miera et al. 2013; Hut et al. 2014; Dardente and Simonneaux 2022)⁠. Despite this opposing coupling between T3 availability and GnRH release between long-day and short-day breeders, it is clear that artificial manipulation of thyroid hormones in seasonal birds and mammals affects seasonal breeding cycles (Woitkewitsch 1940; Follett and Nicholls 1988; Anderson and Barrell 1998; Billings et al. 2002; Anderson et al. 2003; Yoshimura et al. 2003; Yamamura et al. 2006; Barrett et al. 2007)⁠⁠. The mechanism by which thyroid hormones control GnRH release is not completely understood, but effects on hypothalamic neuronal activity, and on the encasement of the axonal terminals of GnRH neurons by tanycytic end-feet has been proposed (Yamamura et al. 2004, 2006; Wood et al. 2015; Dardente and Simonneaux 2022)⁠⁠.

Several lines of evidence suggest a central role of tanycytes in seasonal rhythms of energy metabolism in addition to their role in seasonal breeding (Appenroth and Cázarez-Márquez 2024)⁠⁠. In the Siberian hamster (*Phodopus sungorus*), a mammalian model organism with seasonal rhythm in energy balance, hypothalamic T3 administration triggers responses in body mass and appetite (Barrett et al. 2007; Murphy et al. 2012)⁠⁠. In mice, tanycytes respond to metabolites within the cerebrospinal fluid, such as glucose, suggesting sensitivity to the animal’s energetic state (Frayling et al. 2011; Orellana et al. 2012; Benford et al. 2017; Elizondo-Vega et al. 2019)⁠. Tanycytes also seem to control access of bloodborne substances to the hypothalamus parenchyma via the cerebrospinal fluid and vessel fenestration (Langlet et al. 2013a, b; Balland et al. 2014; Langlet 2014)⁠⁠. Tanycytic processes extend into hypothalamic nuclei related to energy homeostasis, i.e. the arcuate nucleus, ventromedial nucleus and dorsomedial nucleus (Dale 2011; Bolborea and Dale 2013; Pasquettaz et al. 2021; Dali et al. 2023)⁠⁠. Tanycytes are also a hypothalamic stem cell niche and at least a subset of tanycytes-derived neurons migrate to populate the aforementioned hypothalamic nuclei and acquire appetite-regulatory neuropeptidergic phenotypes (Lee et al. 2012a; Haan et al. 2013; Yoo et al. 2021)⁠. The retinoic acid pathway is thought to regulate cell proliferation and many elements of this pathway are known to be under photoperiodic control within tanycytes in seasonal rodents, e.g. Siberian hamster (Melum et al. 2024)⁠ and photoperiodic F344 rats (Ross et al. 2004; Shearer et al. 2012)⁠.

**Fig. 1:**
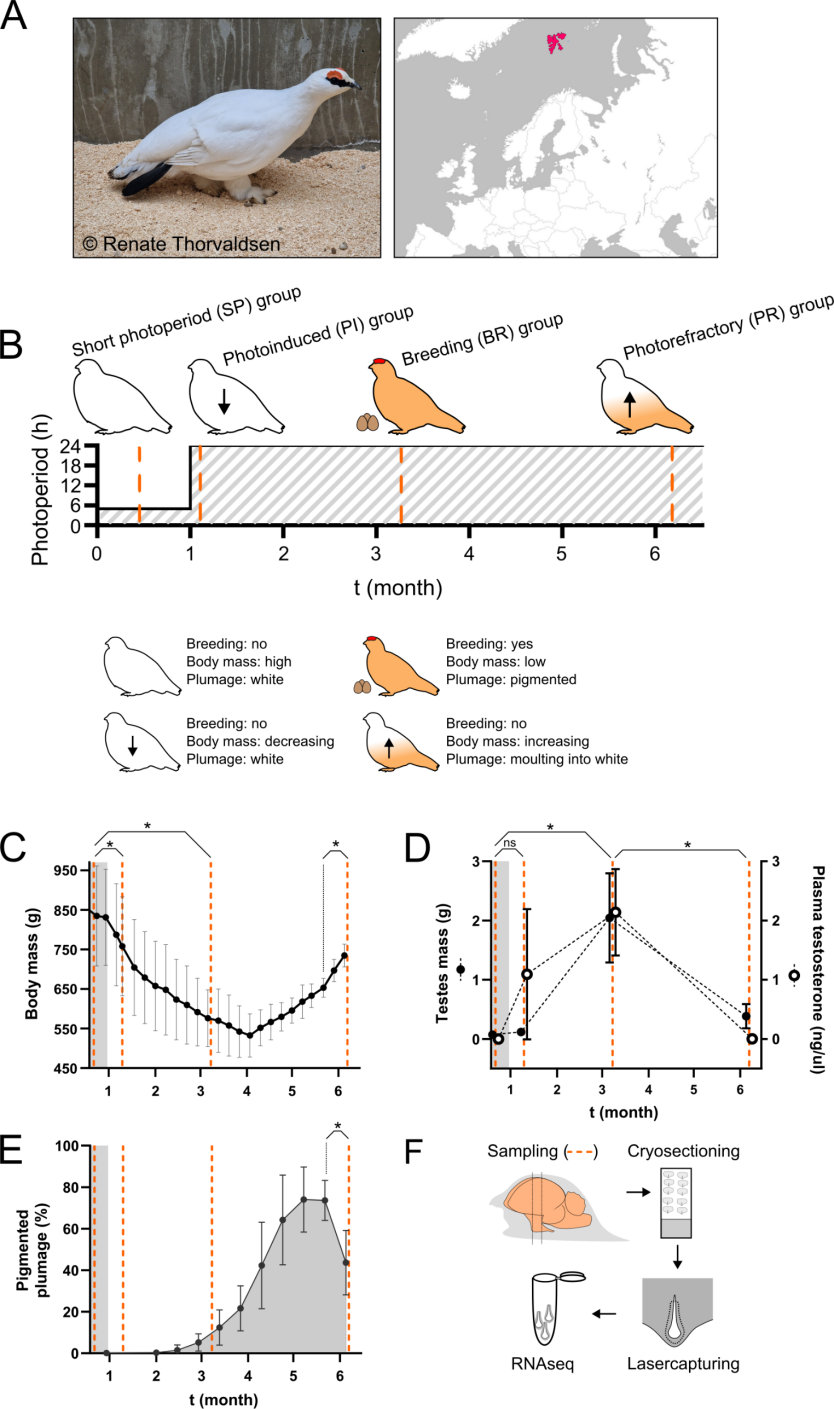
The Svalbard ptarmigan, experimental design and physiological data. (**A**) The Svalbard ptarmigan (*Lagopus muta hyperborea*) is a High Arctic bird population permanently inhabiting the archipelago of Svalbard. Pictures show a breeding male bird kept at the ptarmigan facility of the University of Tromsø (picture taken by Renate Thorvaldsen) and its natural habitat of Svalbard (indicated in red). (**B**) Experimental male birds were kept under short photoperiod under which they express a winter phenotype. The birds were directly transferred into constant light which triggers physiological and metabolic responses which includes initiation of breeding. After prolonged exposure to constant light, the birds became insensitive to the stimulating photoperiod and reversed back into their winter phenotype. This is an endogenous timing process called photorefractoriness. (**C-E**) Throughout the experiment we collected physiological data including body mass, plumage score, testes mass and testosterone levels. All data is displayed as mean ± SD. Statistical significance (*) indicates p-values < 0.05 of Tukey’s tests between the indicated points; ns indicates p-values > 0.05. (**F**) Four male birds were sampled at four occasions as indicated by the orange line in panels B-E. Brains were collected, cryo-sectioned, LASER-dissected for tanycytes and an RNAseq was performed.

Although studies in Japanese quail (*Coturnix japonica*) were the first to describe seasonal hypothalamic thyroid hormone conversion as a key regulatory mechanism of seasonal breeding (Yoshimura et al. 2003; Yasuo et al. 2005; Nakao et al. 2008a)⁠⁠, subsequent research on the role of tanycytes in seasonal metabolism, body mass control and appetite, derives exclusively from studies in mammals (Ebling 2014, 2015; Ebling and Lewis 2018; Helfer et al. 2019; Langlet 2019)⁠⁠. In this experiment, we aimed to characterise changes in the tanycytic region with photoperiod in a highly seasonal bird species. For this purpose, we used captive Svalbard ptarmigan (*Lagopus muta hyperborea*, Sundevall 1845). The Svalbard ptarmigan (Fig. 1A) is a subspecies of the rock ptarmigan and a permanent resident of the archipelago of Svalbard (78°N) (Fuglei et al. 2017)⁠⁠ (Fig. 1A). Svalbard ptarmigan display strong seasonal rhythms in breeding, body mass, appetite and coat colour both in the wild and in captivity (Mortensen et al. 1983; Steen and Unander 1985; Stokkan et al. 1986a, b, 1988, 1995; Lindgård and Stokkan 1989; Lindgård et al. 1995)⁠. The winter phenotype is characterised by reproductive quiescence, accumulation of body fat (high body mass) and white plumage. Exposure to LP triggers the summer phenotype characterised by breeding, depletion of fat stores (low body mass) and transition to a brown summer plumage. In common with many seasonally breeding bird species (Dawson et al. 2001; Watanabe et al. 2007)⁠, Svalbard ptarmigan subsequently develop photorefractoriness with extended exposure to LP and this is marked by termination of breeding, moulting from brown summer into white winter plumage and an increase in body mass, effectively reverting to the winter phenotype (Lindgård and Stokkan 1989)⁠⁠.

Using laboratory-based photoperiod manipulation and the innate development of photorefractoriness we generated four different seasonal metabolic/reproductive states in male Svalbard ptarmigan. We then sampled the ependymal region of the 3rd ventricle of the hypothalamus, using LASER capture microdissection, to generate tanycyte-enriched samples for RNAseq. We confirmed the seasonal modulation of both thyroid hormone and retinoic acid signalling pathways, as described in seasonal mammals, and demonstrated that remodelling of the hypothalamic tanycytic region is likely a conserved response to changing photoperiod.

## Materials and methods

### Experimental procedure

Svalbard ptarmigan used in this experiment were bred from wild birds caught on the High Arctic archipelago of Svalbard (78 °N) (Fig. 1A) and reared at the ptarmigan breeding facility at the University of Tromsø (69 °N). All animals were kept in accordance with EU directive 201/63/EU and licenses provided by the Norwegian Food Safety Authority (Mattilsynet, permit ID: 14209).

For this experiment, a total of 20 male birds were used. Birds were housed in individual cages in light and temperature-controlled rooms. Throughout the experiment, the birds had *ad libitum* access to food and water and the ambient temperature was kept constant (8 ± 3 °C) within the animal’s thermoneutral zone (Mortensen and Blix 1986)⁠⁠. Initially, the birds were kept under SP (LD 5:19) under which they kept a high body mass, white plumage and a non-reproductive state (Stokkan et al. 1995)⁠⁠. After 7 weeks under SP, all birds were directly transferred into constant light (LL). Under those conditions, Svalbard ptarmigan lost body mass, prepared for breeding and changed into brown summer plumage (males change colour only after mating) (Stokkan et al. 1988, 1995)⁠⁠. Birds were kept under LL until they developed photorefractoriness, which is marked by an increase in body mass, a termination of breeding and a moult into white winter plumage despite the fact that the photoperiod is held constant (Lindgård and Stokkan 1989)⁠⁠. The SP to LL treatment was chosen to replicate previous experiments which successfully triggered the desired seasonal phenotypes in Svalbard ptarmigan and were known to be handled well by the experimental birds (Lindgård and Stokkan 1989)⁠⁠⁠.

Body mass was measured once a week. The plumage score was assessed every second week by taking a dorsal picture of the birds and calculating pigmentation with the threshold tool in the image analysis software Fiji (Schindelin et al. 2012)⁠⁠. Birds were euthanized at four sampling points, determined based on the moult score, body mass and reproductive state of the birds (Fig. 1B). After euthanasia, whole brains were removed and snap-frozen on dry ice before being stored at -80 °C. Testes were removed and weighed. Furthermore, blood was taken, centrifuged and blood plasma was frozen. Testosterone concentrations in plasma samples were measured with a Testosterone ELISA kit (MyBioSource.com, MBS9711529) following the manufacturer’s manual. Optical densities were measured with a microplate reader set to 450 nm (GM3500, Promega).

Tukey’s multiple comparison test was used to analyse changes in phenotypic parameters. Data was fitted with a one-way ANOVA for testes weight and testosterone levels and with a mixed model for body mass and plumage score. Graphs and statistical tests for phenotypic parameters were produced with GraphPad (version 10).

### Transcriptomics – ependymal region of the 3rd ventricle

The whole brains of four males from each group were sectioned with a cryostat (CM3050 S, Leica Biosystems) to a thickness of 20 μm. Hypothalamic sections were collected on membrane slides (415190-9081-000, Carl Zeiss) and all slides were frozen at -80 °C until further analysis.

For RNAseq analysis, membrane slides with tissues were thawed for 30 s and stained in a 0.2% Cresyl violet solution containing ProtectRNA™ RNase inhibitor (R7397, Sigma-Aldrich). After staining, slides were dehydrated by dipping them into increasing ethanol solutions containing RNase inhibitor (75%, 96% and 100% ethanol) and air dried. Stained and dehydrated slides were further processed using a LASER capture microdissection microscope (PALM MicroBeam system, Zeiss) and the PALMRobo software (V4.8, Zeiss). The area around the third ventricle, matching *Dio2* in-situ hybridization references from previous studies (Appenroth et al. 2020, 2021)⁠, was LASER-dissected (Fig. S1A) and LASER-catapulted onto the adhesive lids of LCM tubes (415190-9211-000, Carl Zeiss). After all samples were taken, RNA was extracted using the RNeasy Micro kit (74004, Qiagen) following the manufacturer’s manual. RNA samples stored on dry ice were sent to BGI Hong Kong for RNA library prep, sequencing (DNBSEQ PE100, BGI), trimming and data filtering.

The data was further processed with the SAGA supercomputer (HPE Apollo 2000/6500 Gen10, Norwegian research infrastructure services). Raw sequences were mapped to the Rock ptarmigan genome (GCA_023344045.1) (Squires et al. 2023)⁠⁠ using the alignment tool bowtie2 (Langmead and Salzberg 2012)⁠. Reads for each gene were summarised using the rock ptarmigan annotation provided by NCBI RefSeq in combination with the read summarization tool featureCounts (Liao et al. 2014)⁠⁠.

Raw counts were analysed with EdgeR (Chen et al. 2016)⁠ using R implemented in Rstudio (R Core Team 2013)⁠⁠ following the published manual (Chen et al. 2014)⁠⁠. In brief, the data was divided into groups and filtered by setting a threshold of a minimum of 15 counts per million (cpm) in total across all samples and 10 cpm in at least four samples (Chen et al. 2014)⁠⁠⁠. Library sizes were normalized using the trimmed mean of M-values (TMM) method and cpm (counts-per-million) values were calculated.

To analyse cell type enrichment in the samples an analysis outlined in Melum et al. (2024) was conducted utilising a hypothalamic single-cell RNAseq dataset (Campbell et al. 2017)⁠⁠. Campbell et al. (2017) characterized different cell types in the mural mediobasal hypothalamus by single-cell transcriptomics (Drop-Seq), including tanycytes. Key gene IDs for each specific cluster identified by Campbell et al. (FC > 2) were retrieved and matched to the Svalbard ptarmigan expression data. The cpm data was summed gene-wise across all samples and was plotted as mean ± SEM across all genes from each cluster. Hence high values within a certain cluster would indicate enrichment of the respective cell type.

TMM-adjusted cpm data was then fitted into a quasi-likelihood negative binomial log-linear model. Next, ANOVA-like testing was conducted by creating a contrast matrix specifying all pairwise contrasts for all 4 groups (6 combinations) and a QL F-test was run to identify differently expressed genes (DEGs). Hence, 6 possible pairwise comparisons were summarized into a single F-statistic. False discovery rate (FDR) values under 0.01 were considered significant. A heatmap for all significant DEGs was constructed with the heatmap.2 R package based on the Z-score of log2 cpm values. Genes and samples were clustered based on Euclidean distance and the heatmap was sub-grouped into 6 separate groups based on gene expression changes (West et al. 2021)⁠⁠. We then normalized gene expression for each significant gene between 0 and 1 and plotted the mean ± SD for each subgroup, hence obtaining the gene expression profile for each subgroup. For each subgroup, we performed a Gene Ontology (GO) enrichment analysis (Wu et al. 2021)⁠ and assigned the 10 highest hits (in terms of FDR values) to the subgroups. When appropriate the parent GO term was stated rather than the specific term as many genes showed overlaps in closely related GO terms. For each subgroup, several genes of interest were listed.

Last, differential gene expression between the winter phenotype (SP group) and the summer breeding phenotype (BR group) was tested by conducting a quasi-likelihood F-test. Subsequently, significant genes (FDR < 0.05) were matched to significant genes (FDR < 0.05) of an identical DEG analysis from Melum et al. (2024) between SP and LP-adapted male Siberian hamsters. Common genes, i.e. differently expressed in both species were used in GO enrichment analyses. Detailed results of GO enrichment analyses can be found at: 10.18710/M82D10.

An R-markdown document, including a further description of the EdgeR transcriptomic analysis, can likewise be found at 10.18710/M82D10.

## Results

### Photoperiod manipulation results in altered seasonal metabolic and reproductive phenotype

We triggered four distinct seasonal phenotypes in male Svalbard ptarmigan (Fig. 1A) by utilising a photoperiodic extension protocol, i.e. transfer from SP to LL (Fig. 1B) and the ptarmigan’s innate development of photorefractoriness. Phenotypic measurements included body mass (Fig. 1C), testes mass and testosterone plasma levels (Fig. 1D), and plumage pigmentation (Fig. 1E).

Birds under SP (LD 5:19) displayed a clear winter phenotype: white plumage, high body mass and reproductive inactivity (SP group). 10 days after the transfer from SP to LL birds showed photoinduced responses (PI group), including a drop in body mass (*p* < 0.01 compared to SP birds, Tukey’s test) and increasing but non-significantly elevated plasma testosterone levels (*p* = 0.12 compared to SP birds, Tukey’s test). After 10 weeks under LL, all experimental birds were reproductive active (BR group). In males, this was characterised by increased testes mass (*p* < 0.01 compared to SP birds, Tukey’s test) and increased testosterone plasma concentration (*p* < 0.01 compared to SP birds, Tukey’s test). Photorefractoriness developed after 23 weeks in LL (PR group). This was displayed by a return from brown summer plumage into white winter plumage (*p* = 0.02 for comparison between the two last moult scores, Tukey’s test), a steady increase in body mass (*p* = 0.02 for comparison between last body mass and body mass 2 weeks before, Tukey’s test) and a drop in gonad mass and testosterone back to SP levels (*p* < 0.01 compared to breeding levels, *p* > 0.05 compared to SP bird, Tukey’s test).

### Changes in developmental processes, thyroid hormone conversion and retinoic acid metabolism define the response to photoperiod in Svalbard ptarmigan tanycytes

Brains from four males from each group were used for RNAseq analysis (Fig. 1F and Fig. S1A). RNA samples from the tanycytic region were sequenced, aligned and reads summarized. A total of 19 879 unique genes were identified, and 14 399 genes were above the filtering threshold. Our in-silico enrichment analysis based on the Campbell et al. (2017) dataset indicates the enrichment of tanycytes in our samples (Fig. S1B). EdgeR analysis using the ANOVA-like QL F-test identified 618 genes as differently expressed genes (DEGs) (FDR < 0.01) across the four sampling points.

We used unsupervised, hierarchical clustering to sort the DEGs into 6 clusters based on their gene expression dynamics over the experiment. This generated four clusters characterised by intermediate to high expression in the SP state and a decline during LL exposure, and two in which the inverse effect was observed. Within the former group, cluster 3 was noteworthy for showing resurgent expression with the onset of photorefractoriness (PR birds), while in the latter group, cluster 6 was noteworthy for showing a spontaneous downturn in expression in PR birds. Jointly these two clusters of ‘refractory-linked’ genes (20 in total) contributed only 3% of the photoperiod-sensitive transcriptome, making statistically-based GO analysis infeasible, but genes in these clusters have a variety of annotated functions as outlined in Table S1. Here, Sestrin 3 (*Sesn3*) and Pitchfork (*Pifo*) are of note. The former, increased in the SP and PR group, is reported to function in glucose and lipid metabolism (Lee et al. 2012b)⁠ and the latter, decreased in SP and PR state, is involved in ciliation and hence potentially cellular sensitivity (Kinzel et al. 2010)⁠.

**Fig. 2:**
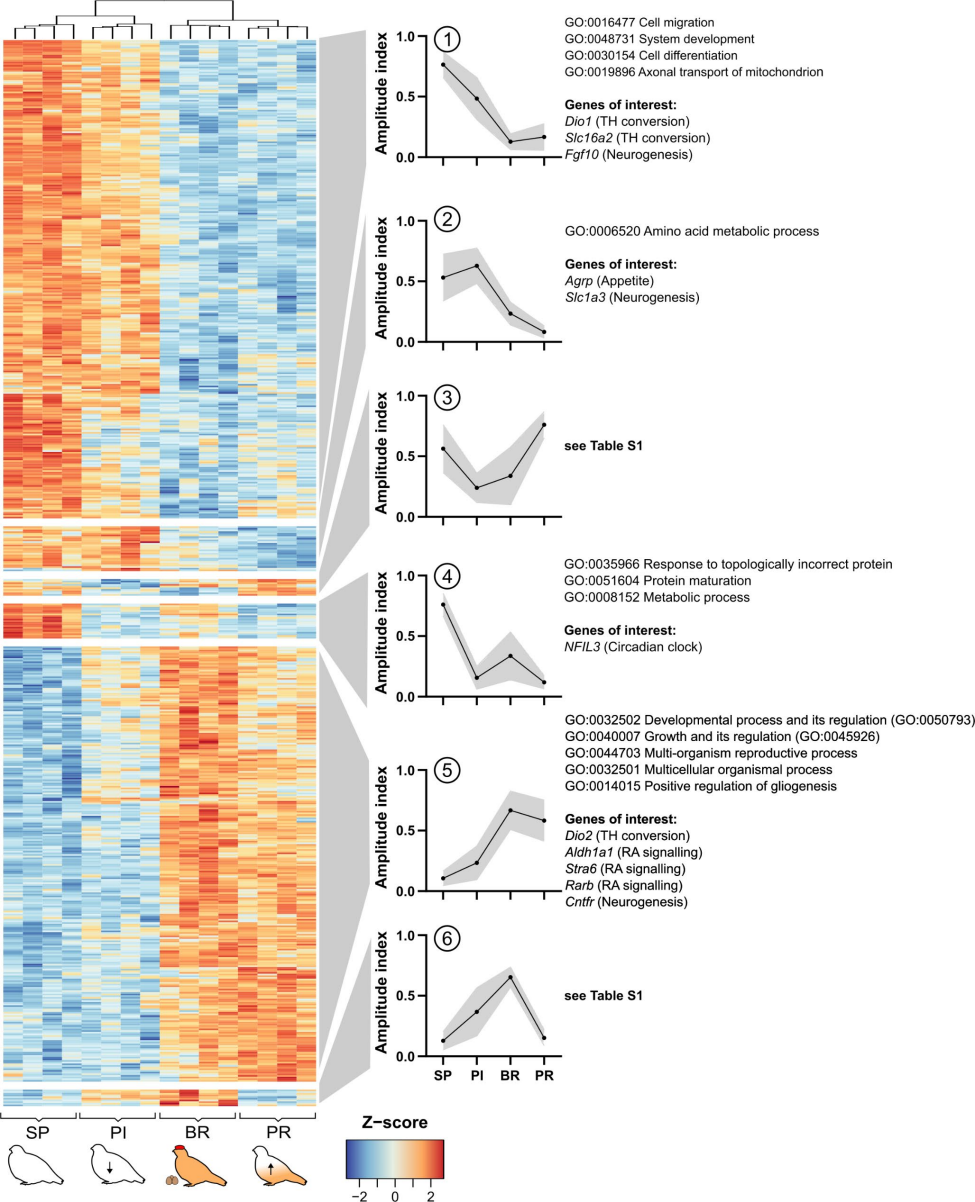
Gene expression profile across the four sampling groups. The heatmap shows all differently expressed genes according to the ANOVA-like QL F-test (FDR < 0.01, 618 genes). The heatmap was divided into six clusters and the expression profile for each cluster is displayed as mean ± SD for all genes of the cluster. GO analysis terms and genes of interest are stated beside each cluster profile. (SP: short photoperiod, PI: photoinduced, BR: breeding, PR: photorefractory, TH: thyroid hormone, RA: retinoic acid)

Clusters 1, 2, 4, and 5 showed no transcriptomic reversal in the PR state back to SP levels, hence can be described as ‘photoperiod-driven’ rather than driven by the endogenous timing mechanism which is presumed to underlie photorefractoriness. We conducted GO enrichment analyses on those ‘photoperiod-driven’ clusters which are summarised in Fig. 2 and Fig. S2.

Some of the genes expressed in cluster 1 (288 genes) are involved in cell migration, cell differentiation and developmental processes. Notably, this cluster also contains genes involved in thyroid hormone conversion and transport (*Dio1* and *Slc16a2* respectively) and tanycyte-derived neurogenesis (*Fgf10*) (Haan et al. 2013)⁠⁠. Cluster 5 (262 genes) also contains a thyroid hormone converting enzyme, *Dio2* and genes involved in the retinoic acid signalling pathway (*Aldh1a1*, *Stra6*, *Rarb*), supporting the enrichment of GO terms relating to involvement in structure development, reproductive processes, regulation of growth and gliogenesis. The remaining clusters (2 & 4) are related to metabolic processes.

In summary, the ‘photoperiod-driven’ clusters form the majority of the expressional response in Svalbard ptarmigan tanycytes and their surrounding cells. This includes genes involved in thyroid hormone conversion, retinoic acid signalling and various developmental processes, including cell differentiation, cell migration and gliogenesis.

### Developmental processes and structural remodelling are conserved features in response to photoperiod

We wished to further explore the conservation of the photoperiodic response in tanycytes between birds and mammals, so we utilised a dataset from the Siberian hamster (*Phodopus sungorus*), which also used the LASER capture technique to enrich for tanycytes from animals kept under SP and LP (equivalent to our SP and BR group, respectively) (Melum et al. 2024)⁠⁠. The Siberian hamster also displays strong photoperiodic cycles in breeding, coat colour, body mass regulation and energy metabolism making it a good comparison to the Svalbard ptarmigan.

**Fig. 3:**
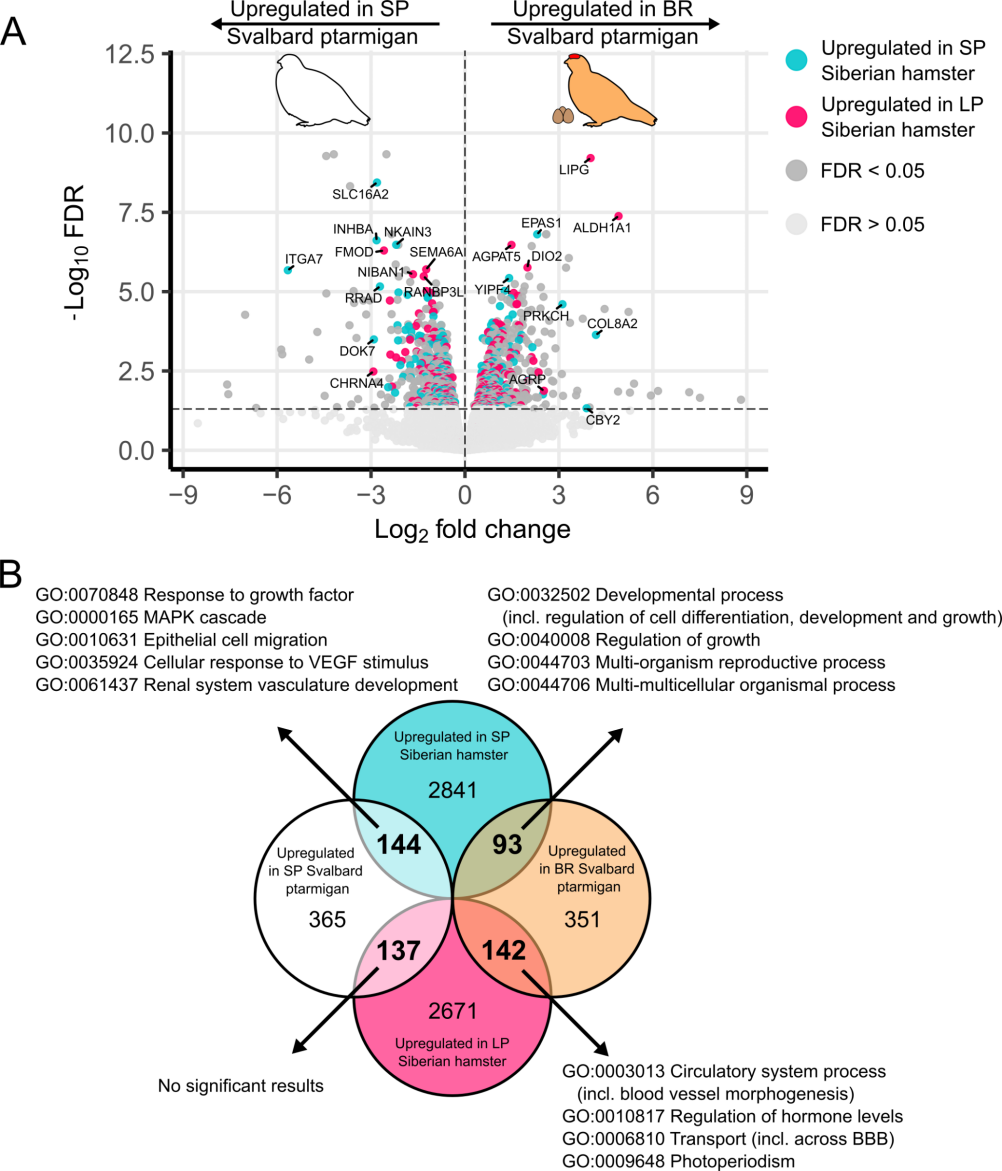
Comparison of photoperiodically controlled tanycytic genes between Svalbard ptarmigan and Siberian hamster. (**A**) 1 232 genes are differently expressed between the winter (SP group) and summer phenotype (BR group) in Svalbard ptarmigan (FDR < 0.05). Of those photoperiodically controlled genes 516 genes are also differently expressed in the tanycytic area of Siberian Hamster (FDR < 0.05) (Melum et al. 2024)⁠⁠. The coloured dots indicate genes which are common in hamster and ptarmigan. Common genes with log2 fold changes higher than 2.5 or -log10 FDR values above 5 are indicated with their respective names. (**B**) GO analyses were performed on all common genes differently expressed in ptarmigan and hamster based on their photoperiodic overlap. The top 10 hits were summarized when appropriate. (SP: short photoperiod, LP: long photoperiod, BR: breeding, FDR: false discovery rate, MAPK: Mitogen-activated protein kinase, VEGF: vascular endothelial growth factor, BBB: blood-brain barrier)

We identified 1 232 DEGs in Svalbard ptarmigan between the SP and BR group (FDR < 0.05, 646 genes up in SP, 586 genes up in BR) and compared these to the DEGs identified between SP and LP adapted hamsters (FDR < 0.05, 3 078 genes up in SP, 2 950 genes up in LP). Homologues of 42% of ptarmigan DEGs were also DEGs in the hamster in response to photoperiod (Fig. 3A). We divided those common DEGs based on the overlaps under which photoperiod they were upregulated (Fig. 3B). Hence, we had four different categories: DEGs upregulated under SP in both species (144 genes), DEGs upregulated under LP/LL in both species (142 genes), DEGs upregulated in SP hamster and BR ptarmigan (93 genes) and DEGs upregulated in LP hamsters and SP ptarmigan (137 genes). We then ran GO enrichment analyses on the different categories (Fig. 3B and Fig. S3) and found enrichment amongst developmental cell processes, response to growth factors, reproduction, circulatory processes, transport (incl. across the blood-brain barrier), regulation of hormone levels and photoperiodism as well as enrichment in pathways known to be active in tanycytes such as vascular endothelial growth factor (VEGF) signalling and the mitogen-activated protein kinase (MAPK) cascade (Langlet 2019)⁠⁠. Detailed results of the GO enrichment analyses can be found under 10.18710/M82D10.

## Discussion

In this experiment, we sought to characterize the tanycytes of an avian species undergoing profound changes in seasonal metabolic state. For this purpose, we used a LASER capture microdissection RNAseq approach in the High Arctic Svalbard ptarmigan.

Our analysis confirms our earlier description of the photoperiodic regulation of hypothalamic thyroid hormone signalling in this species (Appenroth et al. 2020, 2021)⁠. Yet, besides *Dio2*, the present study also identified type I iodothyronine deiodinase (*Dio1*) as a DEG. This finding is intriguing, since transcriptomic datasets in the hypothalamus of the Japanese quail, another Galliformes bird species, do not identify *Dio1* as seasonal DEG (Morris et al. 2020; Majumdar et al. 2023)⁠⁠ and while expression of *Dio1* has been reported in the mediobasal hypothalamus of sheep and goats (Dardente et al. 2022, 2024)⁠ we are not aware of any study reporting *Dio1* to be under photoperiodic control in the mediobasal hypothalamus in either birds or mammals. DIO1 can catalyse outer and inner ring deionisation, hence is capable of activating and deactivating thyroid hormone (Bianco et al. 2002; Lechan and Fekete 2005)⁠⁠. Whether seasonal variation in the expression of *Dio1* affects the ratio of thyroid hormones in the hypothalamus of Svalbard ptarmigan remains to be determined.

Besides thyroid hormone conversion our study considered wider aspects of tanycyte biology in a photoperiodic bird. Retinoic acid signalling in and around tanycytes is known to be under photoperiodic control in a range of mammals such as Siberian hamsters and photoperiodic F344 rats (Ross et al. 2004; Shearer et al. 2012; Melum et al. 2024)⁠⁠⁠, while absent in other species like sheep (Lomet et al. 2018; Helfer et al. 2019)⁠⁠. In the present study, several elements of the retinoic acid pathway were upregulated by the exposure to LL, i.e. *Stra6*,* Aldh1a1* and *Rarb*, which are involved in retinol uptake, conversion of retinol to retinoic acid, and the nuclear receptor-mediated response to retinoic acid, respectively. In the F344 rat, high expression levels of these genes correlated with retinoic acid levels in the hypothalamus (Helfer et al. 2012)⁠, and it therefore is likely that a corresponding increase in retinoic acid activity also occurs in the Svalbard ptarmigan under LL.

Retinoic acid signalling is thought to be involved in stem cell regulation and tanycytes are a known stem cell niche in the mammalian brain (Lee et al. 2012a; Haan et al. 2013; Helfer et al. 2019; Yoo et al. 2021)⁠⁠. Accordingly, GO enrichment analyses of significant DEGs across the four experimental groups indicated roles in cell migration and cell differentiation. We also identified that *Fgf10* was regulated by photoperiod, in tanycytes *Fgf10* has been reported as a marker of tanycytes showing neural stem/progenitor markers in adulthood which then populate the arcuate nucleus (Haan et al. 2013)⁠⁠, therefore having potential roles in appetite and seasonal energy balance (Helfer et al. 2019)⁠⁠.

In order to further investigate conserved features of tanycyte biology which are relevant for seasonal energy balance we compared our present dataset with data from published work on the Siberian Hamster (Melum et al. 2024)⁠, a species with a strong body mass cycle. This comparison reveals a range of common photoperiod-responsive genes and GO enrichment analysis reveals those common genes to be involved in developmental processes including response to growth factors, the regulation of cell differentiation, cell development and cell growth and notably in epithelial cell migration, suggesting a conservation of remodelling in response to photoperiod of the tanycytic region.

Common genes were furthermore involved in responses to vascular endothelial growth factor (VEGF) stimuli and circulatory processes, including blood vessel morphogenesis and transport across the blood-brain barrier. In mammals, modulation of blood vessel fenestration via the tanycytes is hypothesized to act as a gateway of peripheral signals into the hypothalamic parenchyma and is possibly mediated via VEGF signalling (Langlet et al. 2013a; Langlet 2014)⁠⁠. These changes can alter the metabolic feedback to the hypothalamus, presumably an important factor in seasonal changes in body mass and voluntary food intake (Appenroth and Cázarez-Márquez 2024)⁠⁠. Hence, our data may be reflective of similar dynamic changes in hypothalamic blood vessels associated with tanycytes as a seasonal response in a photoperiodic bird species.

Despite inferred commonalities, the photoperiodic direction of change seems less conserved between Svalbard ptarmigan and Siberian hamster. For example, while *Dio2* and *Aldh1a1* are upregulated under LP/ LL in both species, *Epas1* (aka *Hif1*) is upregulated in Svalbard ptarmigan under LL but in Siberian hamster under SP. Notably, *Epas1* is hypothesized to be involved in hypothalamic glucose sensing, regulation of VEGF expression and vascular modulation making it an interesting factor for the above-outlined tanycyte functions (Carmeliet et al. 1998; Zhang et al. 2011; Langlet 2019)⁠. The opposite direction pattern can be observed for 45% of all commonly expressed genes and it is tempting to associate this with the opposed fattening cycles of the two species, i.e. Svalbard ptarmigan fatten under SP while Siberian hamsters fatten under LP (Masuda and Oishi 1988; Stokkan et al. 1995; Ebling et al. 1998; Knopper and Boily 2000; Warner et al. 2010; Melum et al. 2024)⁠⁠. Hence, further comparative work is an opportunity to identify tanycytic genes crucial for seasonal energy metabolism.

Besides investigating biological functions of tanycytes our data set also offers a discussion on the temporal dynamics of tanycytes including their role in endogenous timing mechanisms. Photorefractoriness describes the reversal of a seasonal phenotype by becoming unresponsive to the prevailing photoperiod. In our experiment a surprisingly small number of genes showed expression changes during the transition from the breeding (BR) to photorefractory (PR) state, despite there being a very pronounced shift in the seasonal phenotype (increasing body mass and moulting to white). Interestingly, Lomet et al. (2018) also reported minor transcriptional changes in the mediobasal hypothalamus of ewes during the transition to the LP refractory state.

This paradox might be explained in two ways. One, photorefractoriness is mediated outside or downstream of the tanycytes (García-Fernández et al. 2015)⁠ or two, only a small subset of genes (clusters 3 and 6 of Fig. 2) are responsible for driving the reproductive and metabolic changes. In the present study, we identified two photorefractory genes of note, Sestrin 3 (*Sesn3*) and Pitchfork (*Pifo*) (Kinzel et al. 2010)⁠⁠.

Both genes are involved in glucose metabolism and altered cellular sensitivity to signals. In particular, SESN3 has a role in the sensitization to insulin (Tao et al. 2015)⁠ and changes in the number of primary cilia, potentially mediated by PIFO, can alter signalling through a reduction in G-protein coupled receptor signalling (Schou et al. 2015)⁠⁠. Recently, changes in the number of cilia in response to gestational and post-weaning photoperiod were reported in Siberian hamsters of different seasonal metabolic states (Melum et al. 2024)⁠. This is of particular interest since ciliopathologies can cause severe metabolic phenotypes (Loktev and Jackson 2013; Volta and Gerdes 2017)⁠⁠. While more research is required, it is attractive to speculate that changes in sensitivity to downstream hypothalamic circuits through altered cilia-based cellular signalling in tanycytes could drive photorefractoriness.

In summary, our data strongly suggest a conserved seasonal role of tanycytes between birds and mammals, besides hypothalamic thyroid hormone conversion we found markers of retinoic acid signalling and cell developmental processes. Comparison between Svalbard ptarmigan and Siberian hamster further suggests evolutionary conservation of photoperiodic genes regulating cellular differentiation and dynamic changes in vascularization. However, the modest transcriptome changes seen during the development of photorefractoriness suggest that rather subtle changes in tanycytes occur during the reversal of the summer seasonal state.

## Electronic supplementary material

Below is the link to the electronic supplementary material.

Supplementary Material 1

## Data Availability

R-script, physiological measurements and gene expression data have been deposited in DataverseNO: 10.18710/M82D10.
