# Supplementary material for: Tanycytes from a bird’s eye view: gene expression profiling of the tanycytic region under different seasonal states in the Svalbard ptarmigan
Source: J Comp Physiol A Neuroethol Sens Neural Behav Physiol. 2024 Sep 20;211(1):87–99. doi: 10.1007/s00359-024-01716-3 (PMC11846777; doi:10.1007/s00359-024-01716-3)
Supplement: Supplementary file 1 — Supplementary Material 1 [file 359_2024_1716_MOESM1_ESM.pdf]

Table S1. Differently expressed genes of cluster 3 and 6 and the supposed role in relation to metabolism. Gene function is based on www.genecards.org, other sources are cited appropriately.

Cluster	Gene: Symbol	Gene: full name	Function
3	*Map1b*	microtubule associated protein 1b	Mictrotubule assembly/ Neurogenesis
	Ptms	parathymosin	DNA replication (predicted)
	Proser2	proline and serine rich 2	Unknown
	Sesn3	sestrin 3	ROS regulation, blood glucose regulation, insulin resistance, lipid storage
	Dpysl3	dihydropyrimidinase like 3	Filamin binding activity, actin filament organization, cell projection organization, response to axon injury
	Pmepa1	prostate transmembrane protein, androgen induced 1	Induced by androgens and transforming growth factor beta (TGFB), suppression of Androgen receptors and TGFB pathway
	Abcg1	atp binding cassette subfamily g member 1	Macrophage cholesterol and phospholipids transport, may regulate cellular lipid homeostasis in other cell types
	Cdh6	cadherin 6, type 2, k-cadherin (fetal kidney)	Cell differentiation and morphogenesis, kidney development
	Thbs1	thrombospondin 1	Mediates cell-to-cell and cell-to-matrix interaction
	Il17d	interleukin 17d	Cytokine activity, might be involved in obesity (Geurtsen et al. 2020)
6	*Sec31b*	sec31 homolog b, copii coat complex component	Unknown
	Ube2h	ubiquitin conjugating enzyme e2 h	Degradation of abnormal or short lived proteins
	Ccdc78	coiled-coil domain containing 78	Unknown
	Loc125698117	cholesterol side-chain cleavage enzyme, mitochondrial	Mitochondrial conversion of cholesterol to pregnnenolone
	Fbrsl1	fibrosin like 1	Protein coding gene
	Loc125695489	synaptotagmin-5-like	Thought to bind calcium (Synaptotagmin 5)
	Kdm7a	lysine demethylase 7a	Enables histone demethylase activity; methylated histone binding activity; and transition metal ion binding activity. Involved in histone lysine demethylation.
	Pifo	primary cilia formation	Cilia disassembly (Kinzel et al. 2010)
	Tmem114	transmembrane protein 11	Lens and eye development
	Ccdc33	coiled-coil domain containing 33	Predicted to be active in peroxisome

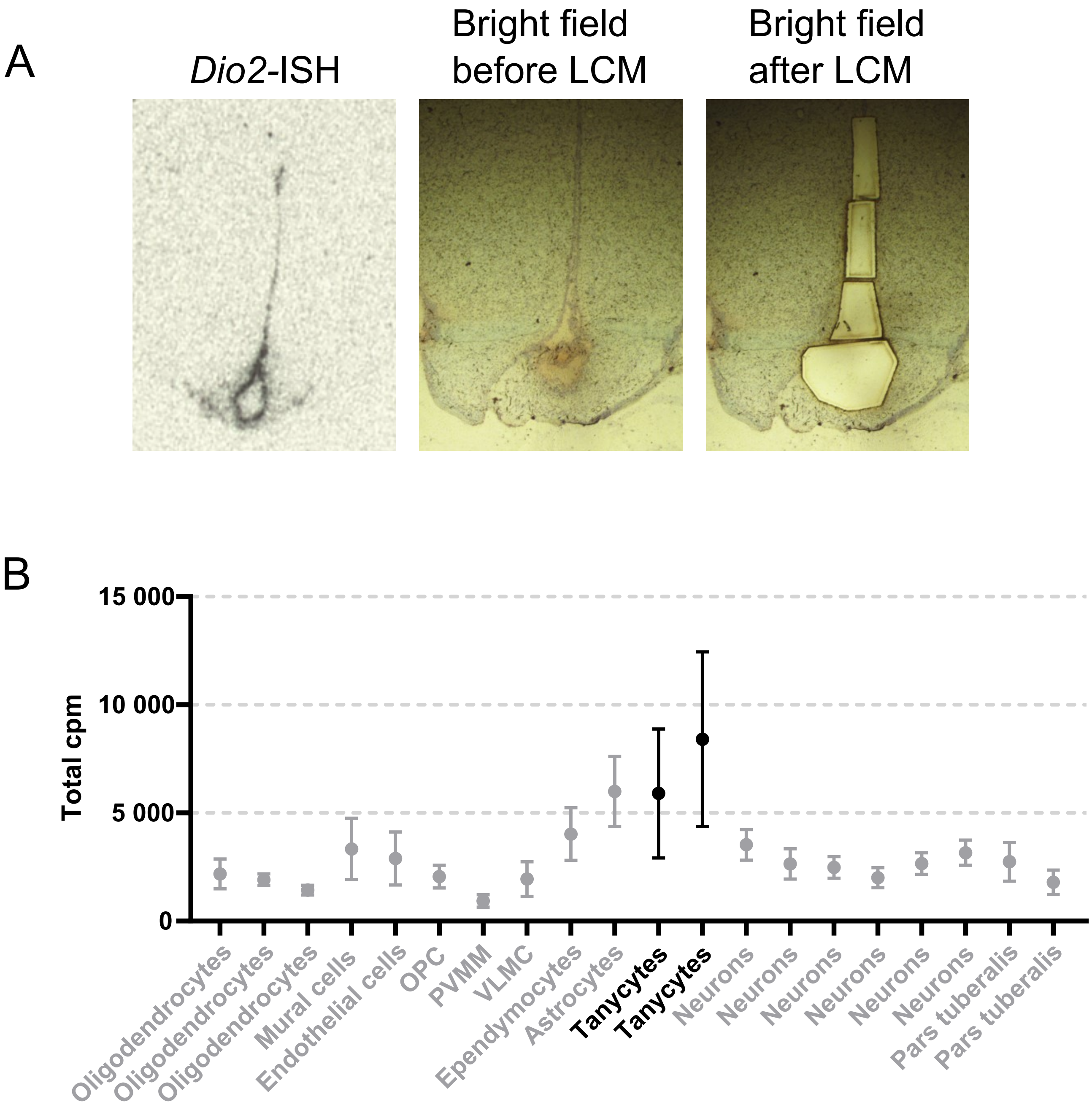

Fig. S1. Tanycytes-enriched samples. (A) Cryo-sectioned brain samples from Svalbard ptarmigan were dissected for tanycytes around the third ventricle, radioactive *Dio2 in situ* hybridization (ISH) was used as reference. Samples were collected by laser-capture microdissection (LCM); subsequently, RNA was extracted and sequenced. (B) *In silico* analysis using a single cell RNAseq dataset of the mural hypothalamus (Campbell et al., 2017) confirms enrichment of tanycytes in our samples. Data displayed as mean± SEM.

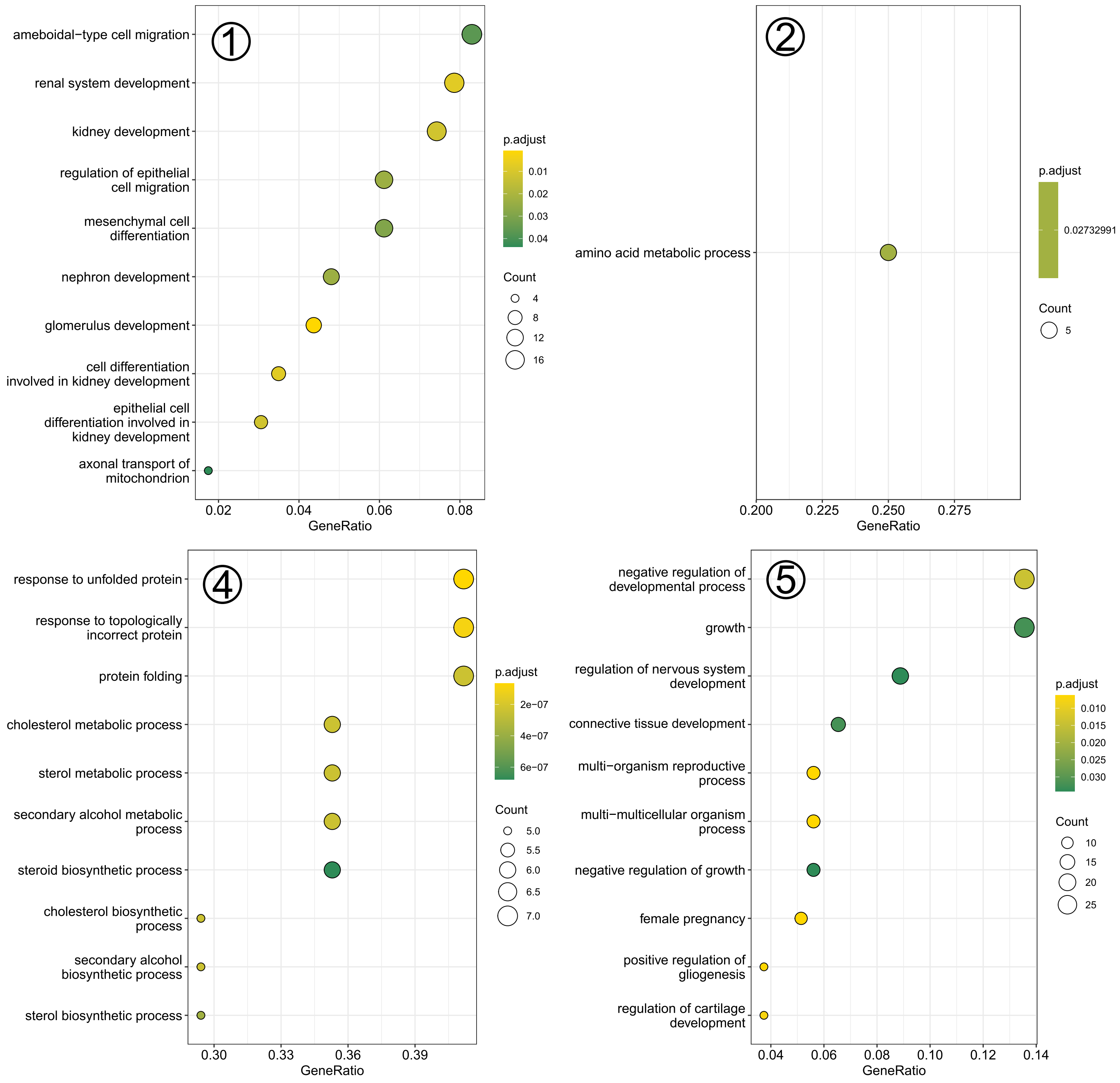

Fig. S2. GO enrichment analysis of differently expressed genes. GO analysis correspond to cluster in **Fig. 2** as indicated by the numbers. Detailed results of GO enrichment analyses can be found under https://doi.org/10.18710/M82D10.

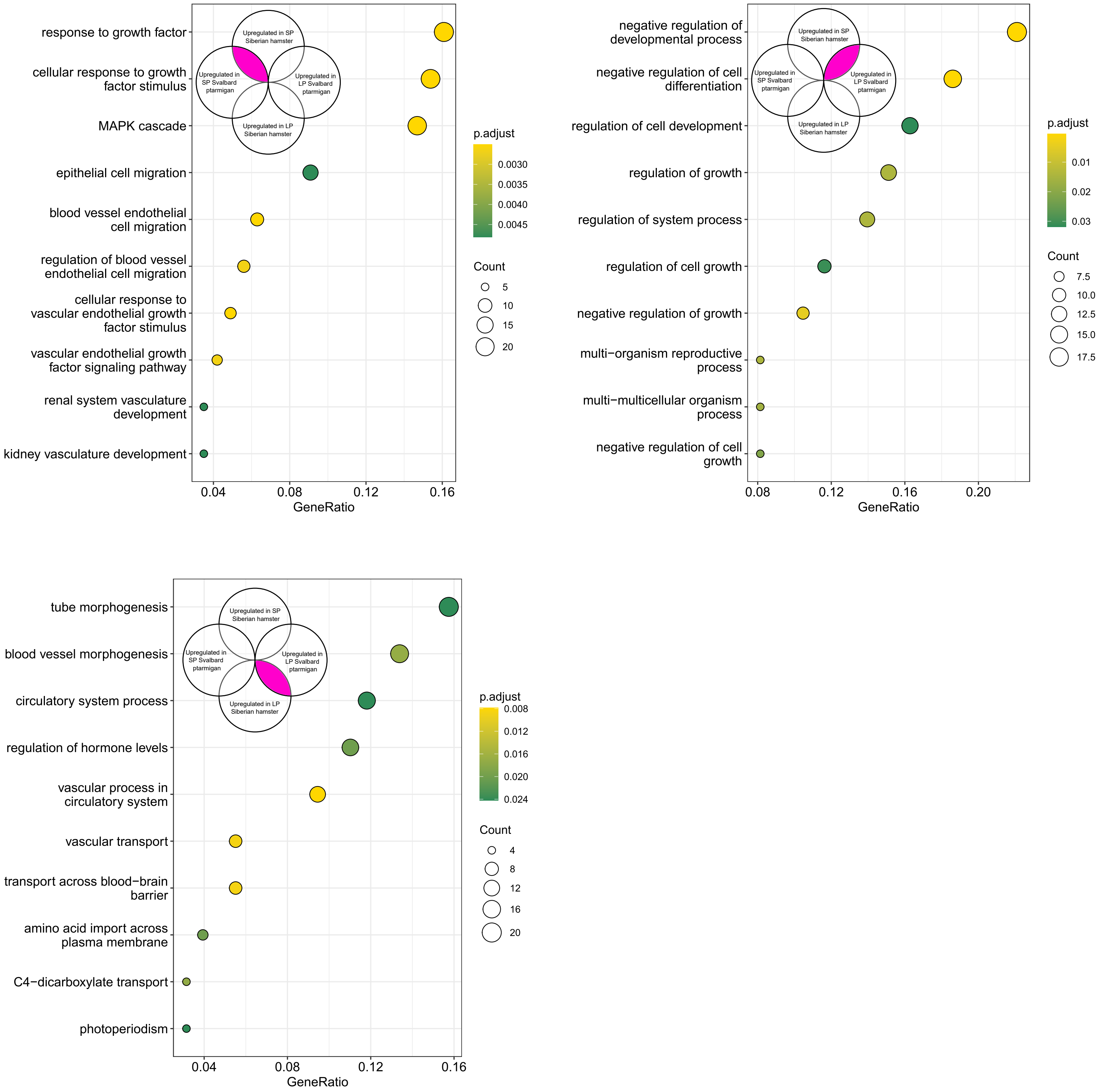

Fig. S3. GO enrichment analysis of common photoperioidc genes in Svalbard ptarmigan and Siberian hamster. Figure corresponds to **Fig. 3B**. Detailed results of GO enrichment analyses can be found under https://doi.org/10.18710/M82D10.
